# Acute reduction of serum 8-iso-PGF2-alpha and advanced oxidation protein products *in vivo *by a polyphenol-rich beverage; a pilot clinical study with phytochemical and *in vitro *antioxidant characterization

**DOI:** 10.1186/1475-2891-10-67

**Published:** 2011-06-15

**Authors:** Boris V Nemzer, Liliana C Rodriguez, Linda Hammond, Robert DiSilvestro, John M Hunter, Zbigniew Pietrzkowski

**Affiliations:** 1 FutureCeuticals, Inc. 2692 N. State Rt. 1-17, Momence, IL 60954, USA; 2 NutraClinical, Inc., 5755 Oberlin Drive, Suite 301, San Diego, CA 92121, USA; 3 Human Nutrition, Ohio State University, Columbus, OH 43210, USA; 4 Applied BioClinical, Inc. 16259 Laguna Canyon Road, Suite 150, Irvine, CA 92618, USA

**Keywords:** polyphenols, beverage, 8-iso-PGF2-alpha, AOPP, NO, antioxidant

## Abstract

**Background:**

Measuring the effects of the acute intake of natural products on human biomarker concentrations, such as those related to oxidation and inflammation, can be an advantageous strategy for early clinical research on an ingredient or product.

**Methods:**

31 total healthy subjects were randomized in a double-blinded, placebo-controlled, acute pilot study with post-hoc subgroup analysis on 20 of the subjects. The study examined the effects of a single dose of a polyphenol-rich beverage (PRB), commercially marketed as "SoZo^®^", on serum anti-inflammatory and antioxidant markers. In addition, phytochemical analyses of PRB, and *in vitro *antioxidant capacity were also performed.

**Results:**

At 1 hour post-intake, serum values for 8-iso-PGF2-alpha and advanced oxidation protein products decreased significantly by 40% and 39%, respectively. Additionally, there was a trend toward decreased C-reactive protein, and increased nitric oxide levels. Both placebo and PRB treatment resulted in statistically significant increases in hydroxyl radical antioxidant capacity (HORAC) compared to baseline; PRB showed a higher percent change (55-75% versus 23-74% in placebo group), but the two groups did not differ significantly from each other.

**Conclusions:**

PRB produced statistically significant changes in several blood biomarkers related to antioxidant/anti-inflammatory effects. Future studies are justified to verify results and test for cumulative effects of repeated intakes of PRB. The study demonstrates the potential utility of acute biomarker measurements for evaluating antioxidant/anti-inflammatory effects of natural products.

## 1. Introduction

Inflammation is associated with and/or implicated in numerous disease states, such as metabolic syndrome, coronary artery disease, diabetes, erectile dysfunction, arthritis, obesity and cancer [1-3]. While inflammation serves as a normal and necessary response to tissue injury and infections, excessive inflammation can be pathological. Dietary components have the potential to modulate inflammatory conditions and play a role in the prevention and/or treatment of disease states [4].

Intake of plant polyphenol-containing foods and products has been associated with beneficial levels of antioxidant/anti-inflammatory markers, and health promoting effects in epidemiological, *in vitro, ex vivo *and *in vivo *studies [4-8]. It is not always clear whether direct, rapid antioxidant/anti-inflammatory effects, or longer-term regulatory actions are responsible. For example, high isoflavone soy protein intake over 4 weeks increases serum radical scavenging capacity based on a total antioxidant status assay; yet adding isoflavones *in vitro *to plasma pooled from subjects samples drawn prior to treatment had little effect [9]. The latter lack of effect occurred even if isoflavones were added well in excess of what oral intake could produce in serum samples. Similarly, a 4-week intake of an isoflavone concentrate can raise erythrocyte contents of the antioxidant enzyme superoxide dismutase 1, but this effect would not be expected to occur immediately after a single intake of isoflavones [10].

In 2001, a NIH working group standardized the definition of a biomarker as "a characteristic that is objectively measured and evaluated as an indicator of normal biological or pathogenic processes, or pharmacologic responses to a therapeutic intervention" [11]. Biomarkers can indicate a variety of health or disease characteristics including responses to various types of interventions such as diet, nutrients, or therapeutics. A biomarker can be classified as a surrogate end-point biomarker (i.e., a marker that is intended to substitute for a clinical end point). Generally, a surrogate end point is expected to *predict *clinical benefit, harm, or lack thereof, on the basis of epidemiological, therapeutic, pathophysiological, or other scientific evidence [12].

Biomarker measurements allow investigation of food and nutritional products in human subjects over a shorter time course as compared to following gross clinical endpoints such as disease occurrence or symptom progression. It also allows researchers to avoid data interpretation and compliance-issues often associated with longer studies.

The present study examines the effect of short-term consumption of a polyphenol-rich beverage (PRB) on several biomarkers related to antioxidant/anti-inflammatory processes. The PRB utilized in this study is called SoZo^®^, and contains numerous fruit and vegetable extracts that are described in detail in the materials and methods section. It includes coffee fruit extract as the first listed ingredient, which is rich in polyphenolic compounds [13,14]. The serum biomarkers that were followed during the study include 8-iso-PGF2-alpha, advanced oxidation protein products (AOPP), nitric oxide (NO), C-reactive protein (CRP) and the hydroxyl radical antioxidant capacity (HORAC) assay. These markers are further described below.

8-iso-PGF2-alpha is an isoprostane derived from arachidonic acid via lipid peroxidation *in vivo*. It is a potent vasoconstrictor, and is considered a gold standard for measuring oxidative stress *in vivo *[15-17]. Studies have shown that serum levels of this biomarker are raised under certain inflammatory conditions such as obesity, diabetes, arthritic and cardiovascular diseases [15,18-21].

AOPPs are the products of plasma protein oxidation--especially oxidation of albumin. Because of its rapid response to changes, it is thought to be suitable for measuring short-term changes in oxidative stress [22]. Serum levels are increased in subjects with inflammatory conditions such as ulcerative colitis, ankylosing spondylitis and renal failure [23,24]. Increased AOPP levels also correlate with cardiovascular disease markers--they may have a causal relationship in the development of atherosclerosis, as has been shown in rabbits [25].

Nitric oxide (NO) is a ubiquitous compound in the body that plays an important role in vasodilation via the relaxation of vascular smooth muscle, and hence in increasing circulation in the body. It also inhibits platelet aggregation and leukocyte activation. A number of plant polyphenolic compounds have been shown to modulate NO levels and/or actions [26,27].

CRP is an acute phase protein that is produced mainly by hepatocytes in response to inflammatory cytokines in the body, and is hypothesized to serve as a marker of cardiovascular disease risk. Increased levels also correlate with type II diabetes, obesity and smoking [3,28]. It is present at very low levels in healthy individuals, and an increased intake of foods rich in polyphenolic compounds is inversely associated with serum CRP concentrations [3,29,30].

This acute single-dose investigational pilot study assesses changes in blood levels of targeted biomarkers indicative of antioxidant capacity, oxidative stress, and inflammation. The approach provides rapid results after the treatment of human subjects with tested material in comparison to placebo.

## 2. Materials and Methods

### 2.1 PRB Composition

The design and formulation of the polyphenol-rich SoZo^® ^beverage (PRB) was based upon a rational examination of existing scientific information regarding potential health benefits of the individual ingredients. The PRB consists of two major components:

1. 1.) A proprietary, powdered blend of selected freeze-dried whole fruit and vegetable powders, concentrated plant phytonutrients, and fruit and vegetable extracts consisting of: whole coffee fruit extract (CoffeeBerry^®^), *Coffea arabica*; calcium fructoborate (FruiteX B^®^), a patented plant-mineral complex; grape seed, *Vitis vinifera*; North American wild blueberry, *Vaccinium augustifolium*; quercetin, *Sophora japonia *L; resveratrol, *Polygonum cuspidatum*; bilberry, *Vaccinium myrtillus *L; raspberry, *Rubus idaeus*; cranberry, *Vaccinium macrocarpon*; prune, *Prunus domestica*; tart cherry, *Prunus cerasus*; strawberry, *Fragaria chiloensis*; grape seed extract, *Vitis vinifera*; broccoli sprouts, *Brassica oleraca italica*; broccoli, *Brassica oleraca italica*; tomato, *Lycopersicon esculentum*; carrot, *Dacus carota satvia*; spinach, *Spinacea oleracea*; kale, *Brassica oleracea acephala*; brussels sprout, *Brassica oleracea gemmifera*; pomegranate extract, *Punica granatum*; and acai pulp, *Euterpe oleracea*. All components of the powdered blend were supplied by FutureCeuticals, Inc. (Momence, IL).

2. 2.) A preservative-free liquid delivery system used to dissolve the previously described powder mixture. This liquid consisted of a blend of juices of grape, *Vitis vinifera*; pomegranate, *Punica granatum*; pear, *Pyrus communis*; apple, *Pyrus malus*; strawberry, *Fragaria chiloensis*; acai, *Euterpe oleracea*; yumberry, *Myrica rubra*; cupuacu, *Theobroma grandiflorum*; and camu camu, *Mycaria dubia*; as well as Sensoril™ (a standardized extract of Ashwagandha (*Withania somnifera*); and natural flavors and colors.

### 2.2 Phytochemical Analysis

#### 2.2.1 Ellagic acid content

The sample was analyzed with an HPLC Agilent 1100 (Agilent Technologies, Palo Alto, CA) equipped with diode array detector. Ellagic acid was detected at 254 nm. Separations were done on a 4.6 X 250-mm C-18 reversed-phase column Waters SymmetryShield RP18 (Milford, MA) containing particles with a size of 5 μm. The mobile phases were 0.1% perchloric acid (mobile phase A) and methanol (mobile phase B). The gradient system began with 50% of the mobile phase A and 50% of the mobile phase B, and was changed to 88% mobile phase B linearly after 20 minutes. After 40 minutes, the column was re-equilibrated to the starting conditions. The retention time of ellagic acid at a flow rate of 1 mL/min was 19.059 min as determined by co-injection with an ellagic acid standard (PhytoLab, Vestenbergsgreuth, Germany).

#### 2.2.2 Total polyphenol content

Total polyphenol content was determined by spectrophotometry according to the Folin-Ciocalteu method, and was calibrated against gallic acid standard (Sigma-Aldrich, St. Louis, MO). Results were expressed as grams of gallic acid equivalents [31].

#### 2.2.3 Total anthocyanin content

Total anthocyanin content was determined by UV-vis spectrophotometry, using malvidin 3-glucosinolate (PhytoLab, Vestenbergsgreuth, Germany) as an external standard. 2% methanolic HCl solution was used for anthocyanins extraction. Samples were sonicated at 50°C for five minutes and shaken vigorously for 30 seconds. The absorbance was read at 535 nm. The amount of total anthocyanins was calculated as malvidin 3-glucoside equivalents.

#### 2.2.4 Chlorogenic acid content

Chlorogenic acid content was determined by UV-vis spectrophotometry Shimadzu 1650PC (Kyoto, Japan) at 325 nm. Samples were prepared using 50% methanol/water solution and sonicated for 5 minutes. Chlorogenic acid from USP was used as an external standard.

#### 2.2.5 Proanthocyanidin content

Proanthocyanidin content was determined using validated dimethylaminocinnamaldehyde colorimetric method [32] originally modified by Brunswick Labs (Norton, MA) using a commercially available A2 dimer standard (HPLC grade; purity >99%; Extrasynthese, France; Cat. # 0985 S).

### 2.3 Antioxidant Analysis

#### 2.3.1 Hydrophilic Oxygen Radical Absorbance Capacity (ORAC) assay

Hydrophilic ORAC assay was performed according to the method of Ou et al [33]. The area under the curve (AUC) was calculated by integrating the relative fluorescence curve. The net AUC of the sample was calculated by subtracting the AUC of the blank. The regression equation between net AUC and Trolox concentration was determined, and ORAC values are expressed as μmol Trolox equivalents (TE) per gram.

#### 2.3.2 Hydroxyl radical antioxidant capacity (HORAC) assay

The HORAC assay was based on a previously reported method [33] modified for the FL600 micro plate fluorescence reader (Bio Tek Instruments, Inc. Winooski, VT). Fluorescein (FL) was used as the probe. The fluorescence decay curve of FL is monitored in the absence or presence of antioxidants, the area under the fluorescence decay curve (AUC) is then integrated, and the net AUC is calculated by subtracting the AUC of the blank from that of the sample antioxidant.

#### 2.3.3 Peroxynitrite radical absorbance capacity (NORAC) assay

The NORAC assay was performed by a modified method [33,34]. Briefly, a stock solution of DHR-123 (5 mM) in dimethylformamide was purged with nitrogen and stored at -80 °C. A working solution with DHR-123 (f.c. 5 M) diluted from the stock solution was placed on ice in the dark immediately prior to the study. Peroxynitrite scavenging by the oxidation of DHR 123 was measured with a FL 600 microplate fluorescence reader (Bio Tek Instruments, Winooski, VT) with excitation and emission wavelengths of 485 and 530 nm, respectively, at room temperature. The background and final fluorescent intensities were measured 5 minutes after treatment with or without SIN-1 (f.c. 10 M) or authentic peroxynitrite (f.c. 10 M) in 0.3 N sodium hydroxide. Oxidation of DHR-123 by decomposition of SIN-1 gradually increased, whereas authentic ONOO- rapidly oxidized DHR-123 with its final fluorescent intensity being stable over time.

#### 2.3.4 Singlet oxygen absorbance capacity (SOAC) assay

Singlet oxygen was generated in ethanol by the molybdate-catalyzed disproportionation of hydrogen peroxide (H2O2) at 37°C. Hydroethidine was used as a probe to singlet oxygen. Hydroethidine was prepared in *N,N*-dimethylacetamide (DMA) to make 40 M solution, 2.635 mM Na_2_MoO_4 _and 13.125 mM H_2_O_2 _working solutions were also prepared in DMA. 125 L of HE solution was added to a well followed by addition of 25 L 2.635 mM Na_2_MoO_4_^2- ^and 25 L 13.125 mM H_2_O_2 _, respectively.

### 2.4 Clinical Study

The clinical pilot study protocol (FC-NC-0902) was approved by the Institutional Review Board at Vita Clinical SA, Avenida Circunvalacion Norte #135, Guadalajara, JAL, Mexico 44270. Males and females between the ages of 45-55 years, with a BMI of 25-36 were recruited for the study. Other inclusion criterion included: subjects otherwise reported themselves as "healthy" (no known disease conditions), were not on any medications, were not allergic to fruits and were not experiencing any digestive problems. A total of 31 subjects were randomized into two groups; a PRB and placebo group.

PRB and placebo samples were prepared in coded tubes and refrigerated at approximately 1.5-3.5°C until time of serving. PRB samples were obtained from a commercially produced lot of the SoZo^® ^beverage. The placebo consisted of a commercially available pear juice that was freshly diluted 1:9 in water. All samples were shaken well immediately prior to administration.

All study participants were informed about study goals, study design and PRB features, and signed a consent form prior to treatment. Study participants were instructed to fast overnight and to avoid vigorous exercise for 12 hours prior to participation. All study participants were routinely asked about how they were feeling during the four hours of post-treatment, in part to identify subjects that might have been experiencing hypoglycemia, so as to avoid collection of data from subjects affected by low blood glucose levels during this acute study. Blood collection was performed by medical laboratory scientists and phlebotomists. Blood was drawn from the median cubital vein using Vacutainers (Becton, Dickinson, Franklin Lakes, NJ). Sample collection was initiated immediately following informed consent, and was performed on all participants at 7:00 AM (T0).

Subjects were randomly given a single 3 oz. dose of PRB or placebo immediately following the baseline blood draw (T0). Subsequent blood samples were collected at 1 (T60), 2 (T120), 3 (T180) and 4 (T240) hours following consumption. Blood was centrifuged at 2000 *g *for 10 minutes and the serum was transferred to fresh tubes for storage at -80°C. Participants were required to remain inactive during the entirety of the experiment and were allowed to drink only water. They were encouraged to write down any subjective observations due to the treatment.

Blood samples of all subjects were analyzed for 8-iso-PGF2-alpha levels using Cell BioLabs assay kit #STA337 (San Diego, CA). Due to the wide range of serum 8-iso-PGF2-alpha levels determined at T0 and the desire to study a population with low-grade inflammation, a subgroup of subjects from both the treatment and placebo groups (10 per group) with the highest 8-iso-PGF2-alpha levels was utilized for further analysis. The subgroup's blood samples were assayed for additional biomarkers, including advanced oxidation protein products (AOPP) using Cell BioLabs assay kit #STA318 (San Diego, CA); nitric oxide (NO) using Biomol/Enzo assay kit #AK-136 (Plymouth Meeting, PA); hydroxyl radical antioxidant capacity (HORAC) assay using Cell Biolabs, assay kit #STA-346 (San Diego, CA) and C-reactive protein (CRP) using Hyphen BioMed assay kit #RK010A (Neuville-Sur-Oise, France).

### Statistical Analysis

Means were compared using a two-tailed Student's *t*-test (Microsoft Excel, Microsoft, Redmond, WA). Statistical significance was set at p < 0.05.

## 3. Results

### 3.1 Phytochemical and Antioxidant Analysis Results

Levels of total polyphenols, total anthocyanins, total proanthocyanidins, ellagic acid, and chlorogenic acid as well as antioxidant capacities were determined and are presented in Table 1.

**Table 1:** Phytochemical and antioxidant capacity analyses of PRB sample per serving size (3.0 fl oz or 90 mL)

Compound/measurement	Amount per serving
Total Anthocyanins	9 mg

Total Polyphenols	360 mg

Total Proanthocyanidins	170 mg

Chlorogenic Acid	225 mg

Ellagic Acid	3 mg

ORAC	10,292 μmole TE

HORAC	31,032 μmole TE

NORAC	643 μmole TE

SORAC	11,430 μmole TE

SOAC	7,457 μmole TE

Total ORAC	60,854 μmole TE

TE = Trolox equivalents

### 3.2 Clinical Study Results

**Figure 1:**
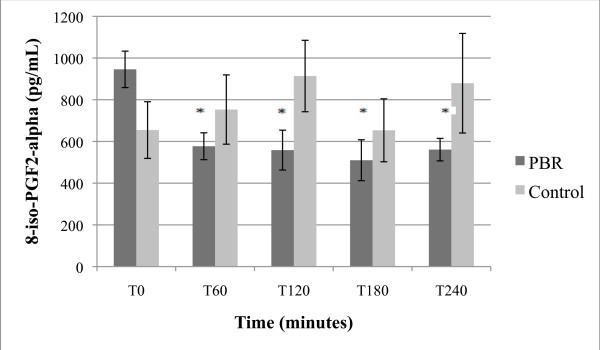
**Effect of PBR on serum 8-iso-PGF2-alpha levels, expressed as pg/mL + SEM**. *p ≤ 0.001 as compared to T0 (paired T-test); and p < 0.05 as compared to placebo (unpaired T-test).

**Figure 2:**
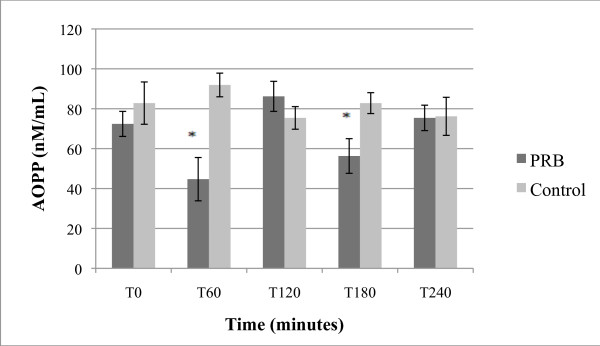
**Effect of PBR on AOPP levels; expressed as nM/mL + SEM**. * p < 0.05 as compared to T0 (paired T-test).

**Figure 3:**
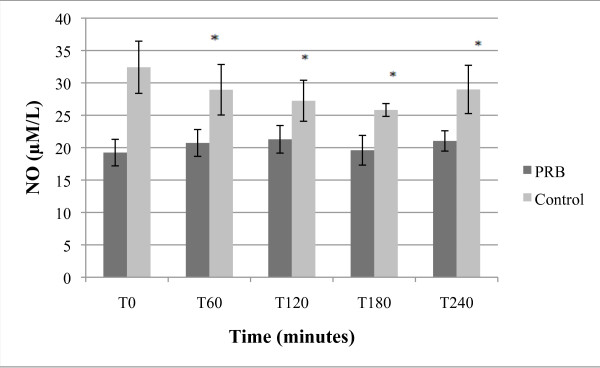
**Effect of PBR on NO levels; expressed as μmoles/L + SEM**.

**Figure 4:**
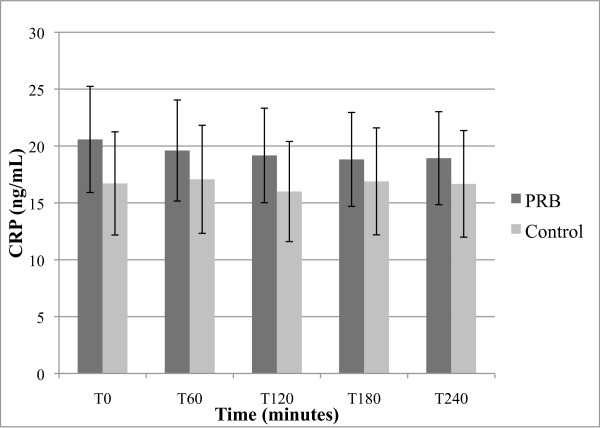
**Effect of PBR on serum CRP levels; expressed as ng/mL + SEM**.

**Figure 5:**
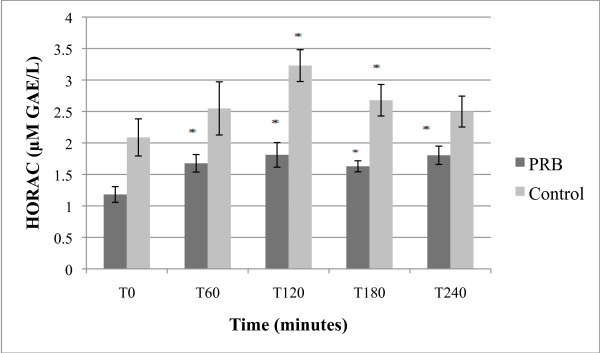
**Effect of PBR on HORAC levels; expressed as μM GAE/L + SEM**. *p < 0.01 as compared to T0 by paired T-test.

The characteristics of the study populations for the randomized, double blind, placebo-controlled acute clinical study were reasonably homogeneous are found in Table 2. Plasma levels of 8-iso-PGF2-alpha at T0 for all subjects are shown in Table 3. Subject number 22 was unable to complete all of the blood sample collections, and hence was not included. Subject 30's samples were disregarded due to hemolysis of blood. Biomarker analyses, performed on the 10 subjects from each group with the highest 8-iso-PGF2-alpha levels, are shown in Figures 1, 2, 3, 4 and 5; results are described below.

**Table 2:** Characteristics of study participants

	Initial Subjects					Subgroup Subjects				
	Mean Age in Years (SD)	Mean BMI (SD)	Females	Males	n	Mean Age in Years (SD)	Mean BMI (SD)	Females	Males	n

**PRB**	48.63 (4.00)	29.47 (3.26)	8	8	16	49.3 (4.72)	30.08 (2.90)	4	6	10

**Placebo**	47.53 (3.09)	28.62 (2.67)	7	8	15	48.5 (3.30)	29.13 (2.51)	5	5	10

SD=standard deviation

**Table 3:** Serum 8-iso-PGF2-alpha levels at T0

Treatment Group Subject Number	8-iso-PGF2-alpha (pg/mL)	Control Group Subject Number	8-iso-PGF2-alpha (pg/mL)
1	1431.2	17	310.9

2	323.7*	18	131.9*

3	946.8	19	1011.5

4	678.7	20	22.1*

5	974	21	1121

6	772.4	23	107*

7	770.9	24	818

8	330.2*	25	206.4*

9	728.4	26	1503

10	965.2	27	414.5

11	308.9*	28	356.4

12	756.9	29	373.4

13	1432.8	30	1190^1^

14	255.2*	31	344.5

15	582.2*	32	294

16	728.4*		

^1^subject data excluded due to hemolysis

*subjects excluded from subgroup analysis due to lowest 8-iso-PGF2-alpha levels

#### 3.2.1 8-iso-PGF2-alpha

As presented in Figure 1, a single dose of PRB resulted in a 40% average reduction of serum 8-iso-PGF2-alpha during first 60 minutes, with depressed values continuing for the next 3 hours. This was statistically significant as compared to both the zero time point (p ≤ 0.001) and the placebo group (p < 0.05). The placebo group had a non-significant increase in 8-iso-PGF2-alpha after treatment.

#### 3.2.2 Advanced Oxidation Protein Products (AOPPs)

As shown in Figure 2, single dose treatment with PRB reduced AOPP blood level by 39% during first 60 min. The decrease was statistically significant (p < 0.05) compared to T0. AOPP values returned to near baseline during the following hours after treatment.

#### 3.2.3 Nitric oxide (NO)

As presented in Figure 3, acute, single dose treatment with PRB increased blood levels of total nitric oxide slightly although the increase was not statistically significant. The placebo group had a statistically significant decrease of NO at T60, which continued to decrease through T180.

#### 3.2.4 C-reactive proteins (CRP)

Baseline blood levels of CRP were moderately high in all study participants, with a mean of 20.6 ng/mL in PRB group, and mean of 16.7 ng/mL in the placebo group. A trend towards decreased CRP was seen after PRB administration (Figure 4), but the decrease was not statistically significant. The PRB treatment mean change from T0 for the average of the time points over the 4 hours post-treatment was just outside the significance range (p = 0.07). Placebo had no significant effect on CRP.

#### 3.2.5 HORAC blood levels

PRB treatment led to significantly increased average blood HORAC values at 1 hour and remained significantly increased over the 4 hours; however, the placebo treatment also produced increases at the T120 and T180 time points (Figure 5). Although the mean increases were greater in the treatment group as compared to the placebo group, differences between placebo and PRB groups were not statistically significant.

#### 3.2.6 Additional testing

Blood serum was also analyzed for general clinical chemistry measurements at T0 and T240. Neither PRB nor placebo adversely affected parameters such as ALT, AST, glucose or LDH levels (data not shown).

No subjective observations were recorded that were considered either positive or negative with regards to the treatment or study design.

## 4. Discussion

Free radicals or reactive oxygen/nitrogen species may play a major role in many diseases, such as cancer, atherosclerosis, Alzheimer's and the entire aging process[35-38]. The most common reactive oxygen or nitrogen species existing in the body are peroxyl (ROO), hydroxyl (HO), hydrogen peroxide (H_2_O_2_), superoxide (O_2_^-^), singlet oxygen (^1^O_2_) and peroxynitrite (ONOO^-^) [39]. As shown in Table 1, PRB showed an especially high *in vitro *antioxidant potential in the hydroxyl radical antioxidant capacity assay.

The phenolic compounds that were found in high concentrations in PRB (Table 1) are some of the most widely distributed plant secondary products. The ability of these compounds to act as antioxidants is well established [40,41]. Additionally, calcium fructoborate, a water-soluble boro-carbohydrate found most notably in certain fruits, nuts, vegetables and legumes [42-44], is a component of PRB, and has been previously reported to exhibit antioxidant and anti-inflammatory activity *in vitro *[45,46].

Acute blood biomarker testing provides a relatively quick and cost effective way to evaluate the potential efficacy of natural products in humans, and has mechanistic screening value. Acute changes in biomarker values suggest that an orally ingested substance: a) survives the chemistries of the gut and/or is converted into active components in the gut, b) is absorbed into the bloodstream, and c) is measurably bioactive, and must work, at least partly, though rapidly accessed mechanisms, rather than by mechanisms requiring repetitive dosing and/or cumulative effects.

The present study demonstrated that PRB has not only antioxidant effects *in vitro*, but that a single intake may impact measurements of serum biomarkers with antioxidant and anti-inflammatory actions in subjects with elevated BMI and 8-iso-PGF2-alpha levels. When the initial evaluation of 8-iso-PGF2-alpha was performed, it was noted that despite selecting for inclusion of subjects with an elevated BMI as a way to investigate subjects experiencing an elevated degree of inflammation and/or oxidation (obesity is linked to low-grade inflammatory states) [47,48]; [49], the levels of serum 8-iso-PGF2-alpha varied widely in subjects at T0 within both experimental groups. Normal serum levels of 8-iso-PGF2-alpha are considered to be 40-100 pg/mL (STA-337-isoprostane-assay-kit). Higher plasma levels are associated with a number of health problems such as cardiovascular disease, diabetes, and inflammatory and arthritic disorders [15,18-21,50]. Chu and colleagues have shown that the plasma 8-iso-PGF2-alpha level in aged rats is 30.6-fold higher than that of young rats, reflecting the enhanced status of oxidative stress in aged animals [51].

Hence, due to the exploratory nature of the pilot study and the desire to observe effects on individuals with elevated serum 8-iso-PGF2-alpha, biomarker analysis in this study was performed on a post-hoc subgroup of the ten subjects from each group with the highest 8-iso-PGF2-alpha levels. The results of post-hoc analyses are of interest and important for generating plausible hypotheses of efficacy [52].

As presented, PRB had statistically significant effects on blood levels of AOPP and 8-iso-PGF2-alpha during the first 60 minutes after the treatment. Additionally, there was a trend toward effects on NO and CRP levels. Both placebo and PRB groups showed a statistically significant increase in HORAC levels. These results suggest that PRB is absorbed and acts quickly in fasting subjects, and raises the possibility that repeated, regular intake could produce effects that may exceed those observed here, with unknown potential clinical benefits.

PRB showed a statistically significant effect of lowering serum 8-iso-PGF2-alpha levels that was rather large; a single intake resulted in a mean reduction of 40% in 60 minutes and an average of 42% over the next 4 hours. This result suggests that the acute effect on plasma 8-iso-PGF2-alpha is both rapid and sustained during first 4 hours after treatment, and justifies further studies using specified 8-iso-PGF2-alpha levels as an inclusion criterion, to both validate this result and explore related effects, such as if PRB may reduce risk of cardiovascular conditions and/or other health problems.

The time course of specific PRB actions was not consistent across all tested markers. The PRB inhibitory effect on AOPP was rather short compared to its effect on PGF2-alpha; the 8-iso-PGF2-alpha effect persisted for at least 4 hours after the treatment, while AOPP readings tended to return to baseline between 1 and 4 hours. This result may be consistent with the literature, as AOPPs are known to respond rapidly to changes [22], although how rapidly they respond is unclear. It is possible that repeated intakes of PRB would lower AOPP readings for more prolonged periods. AOPPs are pro-inflammatory mediators associated with a number of inflammatory conditions [23,24], and also directly impair HDL metabolism, and therefore could play a role in cardiovascular disease development [53].

A number of publications have reported that increases in nitric oxide (NO) levels are beneficial, especially for cardiovascular health [54]. NO can be destroyed by reactive oxygen species, and PRB has antioxidant properties [55]. The PRB-induced increase in NO concentrations was not statistically significant, but the change was different over placebo, as placebo caused NO values to decrease. One possible explanation for the observed post-treatment NO concentrations in the PRB group is that NO values can increase and diminish throughout the day, and that PRB may have prevented one of the NO declines [56,57].

The acute effect of PRB on CRP was not statistically significant. However, the mean acute reduction of CRP at T60 by nearly 5% is nonetheless surprising since blood levels of this biomarker are considered to be rather stable [58]. The placebo group showed a 2.2% increase in CRP at the same time point. Future studies could investigate the potential anti-inflammatory potential of repeated intakes of PRB using CRP as a longer-term marker.

Since PRB was found to have a high HORAC reading during *in vitro *screening (Table 1), it was of interest to determine whether ingested PRB could change HORAC blood values *in vivo *in the clinical study. Hydroxyl radicals play an important role in fatigue and functioning of muscles, muscle respiratory metabolism, muscle inflammation, Alzheimer's disease and DNA damage in general [59-64]. Interestingly, some anti-inflammatory drugs work in part by reducing blood levels of hydroxyl radicals [65].

A single PRB ingestion produced a strong mean rise in HORAC values that was rapid and sustained. Surprisingly, the placebo group also showed a rise in HORAC values. Although the percent increase in the PRB group over T0 exceeded that for placebo for all but the T120 time point (Table 4), the changes were not significantly different between the two treatment groups.

**Table 4:** Percent change from T0 in HORAC levels in clinical study

		% Change over T0			
	**T0**	**T60**	**T120**	**T180**	**T240**

**PRB**	1	55.1 ±7.19*	67.5 ±7.20*	54.5 ±6.52*	75.8 ±9.21*

**Placebo**	1	23.6 ±5.38	74.7 ±5.91*	44.4 ±5.81*	43.0 ±8.47

*p < 0.05 as compared to T0 by paired T-test.

The unexpected rise in serum HORAC values in the placebo group raises a question as to whether extended fasting (up to 16 hours total, as was essentially experienced by subjects by the end of the observation period) may contribute to healthier antioxidant blood status. Data on AOPP, another distinct antioxidant measurement, did not show such a trend in the placebo group. It is therefore possible that only certain types of antioxidant markers change favorably due to extended fasting. Further clinical studies would be required to test this possibility.

When comparing average baseline values (T0) presented in Figures 1, 2, 3, 4 and 5 between placebo and PRB groups, values of 8-iso-PGF2-alpha and HORAC were statistically different in a manner that may be significant. However, average values of AOPP and CRP were not statistically different between the two experimental groups at T0. Since subjects were randomly assigned to each experimental group, this observation was unexpected. A further study using a cross-over design may clarify and substantiate further the results and unexpected observations by allowing for within-subject analysis.

## Conclusions

In conclusion, the collected data demonstrates the potential utility of acute biomarker measurements for evaluating antioxidant/anti-inflammatory effects of natural products such as PRB, and quantitatively shows that a mixture of polyphenol-rich fruit and vegetable components may work acutely on specific oxidative and inflammatory markers in human blood through apparently rapidly acting, though currently unidentified, mechanisms. Results generated by this pilot study justify additional, cross-over type clinical investigations of PRB with narrower inclusion criteria to verify results and to investigate more specific health conditions.

## Abbreviations

AOPP: advanced oxidation protein products; ALT: alanine aminotransferase; AUC: area under the curve; AST: aspartate aminotransferase; BMI: body mass index; CRP: C-reactive protein; DNA: deoxyribonucleic acid; f.c.: final concentration; g: times gravity; GAE: gallic acid equivalents; HCl: hydrochloric acid; HORAC: hydroxyl radical antioxidant capacity; LDH: lactate dehydrogenase,; μmol: micromole; mL: milliliter; nm: nanometer; NO: nitric oxide; ORAC: oxygen radical absorbance capacity; NORAC: peroxynitrite radical absorbance capacity; pg: picogram; PRB: polyphenol-rich beverage; SOAC: singlet oxygen absorbance capacity; T0: zero time point; T60: 60 minute time point; T120: 120 minute time point; T240: 240 minute time point; UV: ultraviolet.

## Declaration of Competing interests

BN and JH are employed by FutureCeuticals, Inc., which provided partial funding for the studies. RD was paid by FutureCeuticals as an independent consultant. ZP and LH are employed by Applied BioClinical, Inc. LR has no competing interests.

## Authors' contributions

BN designed and implemented the biochemical characterization, the in vitro analytical work, and was a co-writer. JH contributed to the study design and to the writing of the manuscript. ZB contributed to the overall study design, the writing of the manuscript, monitoring of the clinical study and direction of the bioclinical analysis. LR was involved in the bioclinical analysis. LH was involved in the bioclinical analysis. RD was involved in the writing of the manuscript. All authors have read and approved the final manuscript.
